# A review on computational models for predicting protein solubility

**DOI:** 10.71150/jm.2408001

**Published:** 2025-01-24

**Authors:** Teerapat Pimtawong, Jun Ren, Jingyu Lee, Hyang-Mi Lee, Dokyun Na

**Affiliations:** Department of Biomedical Engineering, Chung-Ang University, Seoul 06974, Republic of Korea

**Keywords:** biotechnology, machine learning, protein solubility, recombinant protein, solubility prediction

## Abstract

Protein solubility is a critical factor in the production of recombinant proteins, which are widely used in various industries, including pharmaceuticals, diagnostics, and biotechnology. Predicting protein solubility remains a challenging task due to the complexity of protein structures and the multitude of factors influencing solubility. Recent advances in computational methods, particularly those based on machine learning, have provided powerful tools for predicting protein solubility, thereby reducing the need for extensive experimental trials. This review provides an overview of current computational approaches to predict protein solubility. We discuss the datasets, features, and algorithms employed in these models. The review aims to bridge the gap between computational predictions and experimental validations, fostering the development of more accurate and reliable solubility prediction models that can significantly enhance recombinant protein production.

## Introduction

Recombinant proteins are indispensable in biotechnology and bioindustry since they are widely used for various purposes, including disease diagnosis and treatment (Arendt et al., 2016; Demain & Vaishnav, 2009; Morales-Alvarez et al., 2013; Singh et al., 2013), environmental bioremediation (Aer et al., 2024; Wang et al., 2019), industrial bioprocessing (Godawat et al., 2015; Tripathi & Shrivastava, 2019). Despite of the importance of recombinant proteins, approximately 35% of proteins being insoluble and around 25% being soluble but prone to aggregation at high concentrations (Fang & Fang, 2013; Samak et al., 2012). Enhancing solubility significantly improves the functional quality of recombinant proteins and reduces physiological burdens during their production in bacterial hosts by minimizing aggregation, misfolding, and cellular stress (De Simone et al., 2011; Gopal & Kumar, 2013; Xiao et al., 2014). Therefore, enhancing protein solubility is a challenge in the production and use of recombinant proteins in bioindustry (Bhatwa et al., 2021).

To date, numerous strategies have been developed to enhance the solubility of recombinant proteins that are insoluble or prone to aggregation (Ghosh et al., 2004). These strategies include producing proteins at low expression level, optimizing media composition, and incubating at lower temperatures to prevent aggregation (Gutierrez-Gonzalez et al., 2019; Taylor et al., 2017). Another widely accepted approach is to utilize globular and soluble fusion partners such as glutathione-S-transferase (GST) and maltose-binding protein (MBP), which are well-known for enhancing the solubility of fused recombinant proteins (Esposito & Chatterjee, 2006; Nallamsetty & Waugh, 2007). However, these fusion tags are relatively large and consume additional nutrients, thereby reducing recombinant protein production. To address the size issue, short fusion tags have been developed, including small ubiquitin-like modifier (SUMO) (Saitoh et al., 2009), thioredoxin (TrxA) (LaVallie et al., 2000), and short disordered peptides (Ren et al., 2022; Tang et al., 2024). Despite their benefits, these tags are not universally applicable to all recombinant proteins, which necessitates ad hoc experimental trials to identify an effective partner or tag for each specific protein. This creates a significant demand for computational methods that can accurately predict protein solubility, thereby reducing the need for labor-intensive experimental approaches.

Despite the importance of computational prediction of protein solubility, it remains a challenging endeavor due to the complex nature of proteins and the multitude of factors influencing their solubility (Hou et al., 2020; Yang et al., 2021). Advances in high-throughput experimental techniques have enabled the accumulation of extensive protein solubility data across multiple species (Velecky et al., 2022). This accumulating data paves the way for developing computational models capable of predicting solubility based on protein sequences and structures (Habibi et al., 2014).

In this review, we introduce computational methods for predicting protein solubility, emphasizing the principles behind these techniques and highlighting accessible online resources. We discuss the advantages and limitations of the approaches.

Machine learning (ML), a subset of artificial intelligence, enables algorithms to identify patterns in data without explicit programming. In the context of protein solubility prediction, ML is primarily applied through supervised learning, where models are trained on labeled datasets containing known solubility outcomes (i.e., soluble or insoluble proteins). These models, once trained, can generalize to predict solubility for unseen proteins based on input features. The core objective of these models is to achieve predictive accuracy by learning from the underlying data structure while mitigating issues such as overfitting, which can compromise generalization to new datasets.

**Fig. 1.**
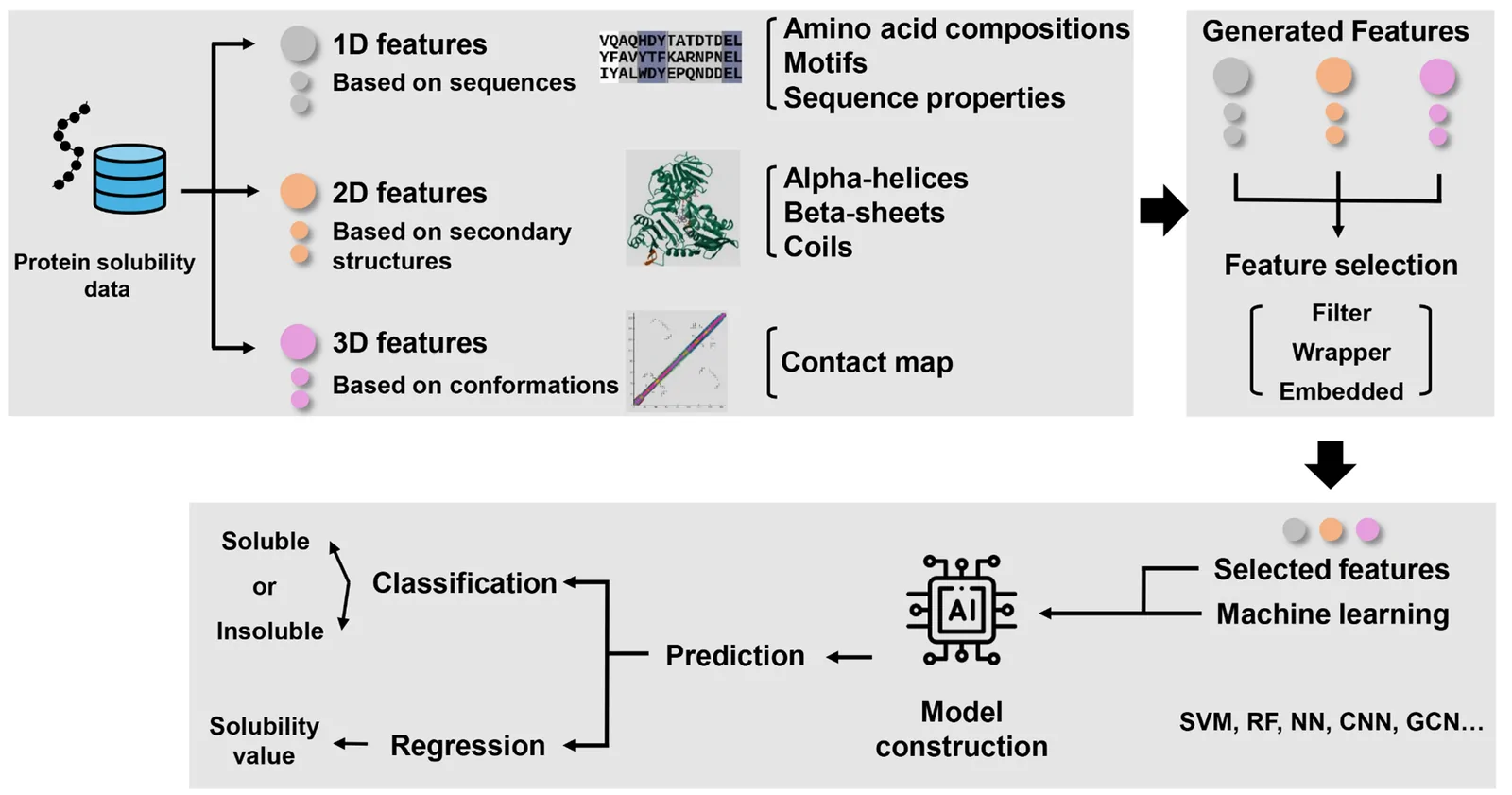
Schematic illustration depicting the general workflow of machine learning-based approaches for predicting protein solubility. This workflow encompasses various stages, including data collection, identification of feature types, feature extraction, feature selection, model training/testing, and the final solubility prediction.

Fig. 1 illustrates the general workflow involved in machine learning-based protein solubility prediction. The process commences with data collection from curated protein solubility datasets. Following this, feature extraction takes place, where relevant attributes (features), such as sequence information or structural properties, are computed. These extracted features are then used during model training, where the algorithm learns from the training data. Finally, the trained model undergoes evaluation and is applied to predict the solubility of novel proteins. This figure encapsulates the iterative nature of the process, highlighting the critical stages necessary for building and validating a predictive model while addressing common challenges like overfitting.

## Computational Models for Protein Solubility Prediction

Developed solubility prediction models to date are summarized in Table 1, including their approach, datasets used for training and testing, features, and employed learning algorithms, their performances, and their availability. Most prediction methods are classification models that determine whether a given protein is soluble or insoluble (Habibi et al., 2014). However, owing to the advance of high-throughput experiment techniques and accumulating data, regression models capable of predicting absolute solubility, such as soluble fraction or percentage, have been developed (Han et al., 2019, 2020).

Solubility prediction models utilize primarily sequence-derived features such as residue compositions, conserved sequence patterns, physicochemical properties calculated from sequences, etc. Recent models also utilize the features obtained from protein 3D structures due to accumulating data of protein structures and advances in structure prediction methods (Hou et al., 2020), including secondary structures, solvent accessibility surface area, backbone torsion angles, residue contact map, etc., which offer insights into residue interactions and their impact on solubility (Chen et al., 2021; Hou et al., 2020).

In the following sections, we will introduce the datasets used for learning prediction models, the features used for learning, developed models and their applications in bioindustry, and challenges in developing solubility prediction methods.

## Datasets for Model Development

For accurate model learning, it is necessary to compile a large amount of protein solubility data. Conventional small-scale random mutagenesis experiments provide detailed insights into how individual mutations affect protein solubility, offering mechanistic understanding for protein engineering (Tachioka et al., 2016). However, these low-throughput experiments are labor-intensive and time-consuming, have inherent bias due to the focus on specific mutation positions and types, and thereby have limited generalizability.

Databases containing protein expression information can provide whether proteins are soluble or not, because well-expressed proteins are mostly soluble. Qualitative solubility data have been compiled by inferring protein expression status from protein data bank (PDB) and target registration database (TargetDB), which have annotations about protein expression (Burley et al., 2019; De Cesco et al., 2020). Basically, most proteins deposited in PDB are soluble and these proteins can be extracted using the annotation about the expression system used. TargetDB is a database in which diverse biological information on therapeutic target proteins are aggregated (De Cesco et al., 2020). This database also tracks the progress of protein targets through various stages of production and structure determination, and serves an annotation of protein status whether soluble or not. Qualitative solubility data over 10,000 have been collected from these two databases and have been utilized for learning models classifying whether a protein is soluble or not. For the accurate development of protein solubility prediction models, it is essential to compile extensive and diverse solubility datasets. Such datasets enhance the model’s ability to generalize across a wide variety of protein sequences, thereby improving the accuracy of solubility predictions. The PDB and TargetDB provide valuable qualitative solubility data inferred from protein expression statuses.

Similarly, TargetTrack database contains information on experimental expression state of proteins, which implies solubility (Berman et al., 2017). The peptide crystallization database (pepcDB) is another database, which compiles empirical data from individual experiments focused on the crystallization of peptides and small proteins (Kouranov et al., 2006). The pepcDB stores target and protocol information contributed by Protein Structure Initiative centers as well as target proteins deposited in the TargetDB. The pepcDB database compiles over 80,000 proteins with solubility information (Kouranov et al., 2006). Qualitative protein solubility data extracted from these databases have been instrumental in developing solubility prediction classifiers. They help mitigate bias from imbalanced datasets, where certain classes of proteins (e.g., soluble vs. insoluble) are overrepresented, and improve model validation when applied to new, unseen data.

Despite the large number of qualitative solubility data and developed classifiers based on the data, now there is a high demand for models capable of predicting quantitative solubilities for enhanced protein engineering. Recent advances in high-throughput methods, especially cell-free methods, have enabled to test a large number of proteins in parallel, allowing for generating expansive datasets that help capture the correlation between protein sequence and solubility. The eSOL dataset is a comprehensive collection of solubility data for *E. coli* proteins (Delaney, 2004). The whole open reading frames (ORF) of *E. coli* were individually amplified by PCR, systematically expressed using the in vitro translation system (PURE system) (Cui et al., 2022), and their solubility were measured at a high-throughput scale. The collected data provide comprehensive solubility profiles for many proteins. This quantitative data is invaluable for developing predictive models that assess not only whether a protein will be soluble but also the degree of its solubility. Such precision enhances the model’s performance, particularly in distinguishing subtle differences in solubility among closely related proteins. Furthermore, quantitative datasets enable advanced feature selection techniques that identify the most relevant features influencing solubility, which is essential for constructing a robust model.

By leveraging these datasets, machine learning models can achieve greater accuracy in predicting solubility across various protein sequences and types. This reduces the reliance on trial-and-error experimental testing, facilitating more efficient protein production in biotechnological applications.

## Features and Feature Calculation Tools

For model learning, diverse properties (features) at residue level or protein level should be generated from sequences, and the features highly correlated with protein solubility are used by learning algorithms to determine whether a protein is soluble or not, or the percentage of solubility.

There are many different sequence-based features that can be calculated or predicted from protein sequences. The simplest features extracted from sequences are the compositions of amino acids, dipeptides, and tripeptides, and the content of charged residues, turn-forming residues, etc., since soluble proteins have statistical preferences to certain residues (Cao et al., 2013, 2015; Chen et al., 2022; Pande et al., 2023; Xiao et al., 2015). Physicochemical properties including hydrophobicity, net charge, etc. can be also calculated from protein sequences. Of diverse physicochemical properties, hydrophobicity or aliphatic index gauges the tendency of proteins to repel water, thereby influencing solubility (Grossmann & McClements, 2023). Evolutionary conserved sequences represented by position-specific scoring matrix (PSSM) or hidden Markov model can be also used as features since like the residue preference there are conserved regions or sequences in proteins that are more frequently found within soluble proteins (Bystroff & Krogh, 2008; Wang et al., 2017). While sequence-based features offer foundational insights into protein solubility, structure-based features provide essential contextual and detailed information. The spatial arrangement of amino acids affects their interactions with each other and with solvents, which in turn directly influences protein solubility (Aguirre-Plans et al., 2021). This relationship improves the accuracy and depth of solubility predictions. For example, protein globularity is one of the structural features that has been correlated with solubility. Globular proteins, e.g., glutathione S-transferase and maltose-binding protein, are highly soluble and are commonly used as a fusion partner to solubilize recombinant proteins (Nallamsetty & Waugh, 2007). In addition to globularity, many diverse features can be obtained from protein structures. Secondary structure contents (helix, sheet, and loop), backbone torsion angles, protein contact map, etc. are also important features that determine protein 3D structure and internal residue interactions, and thereby affect solubility (Hou et al., 2020; Kuhlman & Bradley, 2019). B-factor representing atomic mobility and flexibility gives an insight into how proteins maintain solubility (Sun et al., 2019). Solvent accessibility, accessible surface area, solvation energy, etc. represent interactions with water molecules, determining solubility (Durham et al., 2009).

These structure-based features require precise protein structures to accurately determining atomic level property calculations, which hampered the development of structure-based prediction models since structure determination is biologically laborious process and difficult to obtain large number of protein structures (Chen et al., 2021). However, owing to recent advances in computational protein structure prediction methods, highly accurate protein structures are now easily obtained in silico (Liu et al., 2022; Pak et al., 2023; Ruff & Pappu, 2021). While these methods offer valuable insights, they may not fully capture the complexity of experimentally derived structures, particularly in regions where flexibility or disorder affects solubility (Chen et al., 2021; Hou et al., 2020). Therefore, it is crucial to account for potential discrepancies in models trained on predicted data to prevent error propagation. Integrating both high-quality experimental data and predicted structures is essential for improving the robustness and accuracy of solubility prediction models (Jumper et al., 2021; Liu et al., 2022; Ruff & Pappu, 2021). This combined approach leverages the precision of experimental data alongside the scalability of in silico methods, allowing for more comprehensive datasets that enhance the reliability of machine learning models in protein solubility prediction.

Meanwhile, due to the tedious and repetitive nature of feature calculation, numerous computational tools have been developed to facilitate this process, allowing users to easily download and utilize them for extracting a wide range of features. Available tools for feature calculation are listed in Table 2. Specifically, PROFEAT computes a wide range of protein features, including residue compositions, physicochemical properties, and secondary structures (Zhang et al., 2017). These biochemical characteristics allows to predict various aspects of protein function and behavior. Additionally, tools such as iFeatureOmega, Pfeature, protr, Propy, and Rcpi generate a comprehensive set of sequence-based features including PSSM (Cao et al., 2013, 2015; Chen et al., 2022; Pande et al., 2023; Xiao et al., 2015). POSSUM generates and analyzes PSSMs from protein sequences, aiding in the identification of conserved motifs and functional sites within proteins (Wang et al., 2017). PDBparam computes detailed properties based on protein 3D structure, including charge distribution, accessible surface area, hydrophobicity patterns, secondary structure elements, structural flexibility, and inter-residue contact distances, etc. (Nagarajan et al., 2016). More accurate protein solubility prediction models can be developed with ease by utilizing these publicly available tools and their calculated diverse features.

## Overview of Computation Models, Algorithms, and Features for Protein Solubility Prediction

Protein solubility prediction models reported to date are summarized in Table 1, and briefly introduced in the following subsections. The PROSO, PROSO II, SOLpro, DeepSol, PaRSnIP, NetSolP, PROTSOLM, PLM_Sol, DeepSoluE and SoluProt are classification models that are able to determine which proteins are soluble and which are insoluble. SOLart (Hou et al., 2020), Han et al.'s (2020) Support Vector Regression (SVR) model, and GraphSol (Chen et al., 2021) are regression models estimating the precise numerical value of protein solubility, providing quantitative predictions, which can be useful for fine-tuning protein sequences for industrial applications.

To better interpret the performance of these models, it is important to contextualize common evaluation metrics. Accuracy measures how often the model correctly predicts solubility. Scores above 0.70 typically indicate strong model performance. For instance, DeepSol achieved an accuracy of 77%, reflecting its high predictive reliability (Khurana et al., 2018). However, models with accuracy around 0.60, like SoluProt with 58.5% accuracy (Hon et al., 2021), can still be valuable depending on the dataset and application.

Another important metric is the Matthews Correlation Coefficient (MCC), which assesses the balance between true positive and false positive rates. An MCC value greater than 0.50, such as DeepSol’s 0.55 (Khurana et al., 2018), suggests balanced performance, whereas lower MCC values, like SoluProt’s 0.17 (Hou et al., 2020) may still hold relevance in specific tasks, such as sequence prioritization.

For regression models, R^2^ values between 0.40 and 0.60 are typical due to the complexity of protein solubility prediction (Chen et al., 2021; Hou et al., 2020). These values represent how well the model can explain the variance in protein solubility, making these models useful for fine-tuning protein engineering strategies.

The computational models described in this review employ a variety of algorithms and datasets, providing a comprehensive exploration of different approaches to predict protein solubility. Each algorithm has its strengths and weaknesses in capturing patterns within data. For instance, while some algorithms like support vector machines (SVM) excel at handling non-linear relationships, others, like neural networks, are better suited for capturing complex interactions within high-dimensional data. This diversity enables models to capture distinct patterns from the input data, thereby allowing for generalization across a wide spectrum of protein sequences. The models like PaRSnIP and DeepSol leverage different machine learning techniques such as gradient boosting machines and convolutional neural networks, respectively, which process features in unique ways.

The use of varied datasets also enhances model generalizability. Diverse training data enables models to predict solubility across a broader range of protein sequences, thus improving their robustness and applicability to real-world scenarios. However, despite the potential advantages of changing algorithms and datasets, the inherent complexity of protein solubility prediction may limit the extent to which accuracy scores can be improved. Consequently, model performances often plateau even with different algorithms and datasets. For classification models, accuracy scores typically range between 0.72 and 0.77, with MCC values stabilizing around 0.5. For regression models, R^2^ values typically fall between 0.4 and 0.6, reflecting the inherent difficulty in modeling the quantitative aspects of solubility.

To achieve substantial improvements in predictive performance, especially beyond these thresholds, more diverse and comprehensive datasets are essential. Additionally, incorporating higher-order features, such as structure-based properties, could further refine these models. However, until such datasets and features become widely available, substantial advancements in accuracy, MCC, or R^2^ are unlikely, regardless of algorithmic improvements.

**PROSO, PROSO II, SOLpro :** PROSO, SOLpro, and PROSO II, developed a decade ago, are classification models designed to distinguish between soluble and insoluble proteins (Magnan et al., 2009; Smialowski et al., 2007, 2012). PROSO was trained on *E. coli* dataset compiled from TargetDB and PDB, which included over 14,000 proteins. SOLpro was trained using the dataset of 17,408 *E. coli* proteins, which were collected from PDB, SwissProt, and TargetDB, and the dataset of Idicula-Thomas and Balaji (2005). PROSO II is an updated version of PROSO and was trained on 82,299 proteins, a merged dataset of PROSO’s and the proteins from pepcDB database.

The three models utilized sequence-derived features for learning, including compositions (amino acid, dipeptides, and tripeptide), physicochemical properties (hydropathy, charge, molecular weight, aliphatic index, etc.), secondary structure composition, exposed residues, number of domains, etc. PROSO and SOLpro were initially built based on the chained models of SVM but different in the output layer, naïve Bayes in PROSO and SVM in SOLpro. PROSO II was built based on the chained models of a Parzen window model and a logistic regression classifier with an output model of a logistic regression classifier. The accuracies of PROSO and SOLpro were 0.72 and 0.74, respectively, when crossvalidated. The accuracy of PROSO II on an independent test dataset was 0.75.

PROSO has been successfully applied in experimental settings to predict protein solubility. In validation tests involving 31 mutational variants of two different proteins, FGFR1 oncogene partner (FOP) and centrosome-associated protein 350 (CAP350), PROSO accurately predicted the solubility states for the majority of variants. These solubility predictions were based on experimental factors such as maximum achievable concentration, stability in solution, and the propensity to form aggregates. The model’s predictions aligned closely with experimental results, demonstrating its effectiveness in reducing reliance on trial-and-error approaches in wet-lab experiments.

PROSO is recognized for its computational efficiency and straightforward implementation, making it a practical option for large-scale solubility screening. However, its exclusive reliance on sequence-derived features limits its predictive capacity, particularly for proteins where solubility is influenced by higher-order structural properties. PROSO II, while enhancing performance through expanded datasets and optimized algorithms, continues to face challenges due to the lack of structural data integration, which can reduce its effectiveness in accurately predicting solubility in structurally complex proteins. SOLpro addresses some of these limitations by employing a more advanced SVM-based architecture, which improves predictive accuracy over earlier models. Nevertheless, like PROSO and PROSO II, SOLpro remains constrained by its dependence on sequence-based features, limiting its applicability in scenarios where solubility is heavily modulated by 3D structural conformation and interactions.

**PaRSnIP and DeepSol :** PaRSnIP and DeepSol are classification models designed to predict soluble proteins (Khurana et al., 2018; Rawi et al., 2018), which were trained using an *E. coli* dataset of 69,420 proteins, originally derived from the dataset used to train PROSO II, but with a different preprocessing step. Both models were trained using similar features including sequence length, molecular weight, fraction of turn forming residues, average hydrophathicity, compositions of amino acids, dipeptides, and tripeptides, and secondary structures, exposed residues, etc. The main difference is the learning algorithm they employed: PaRSnIP employed Gradient Boosting Machine (GBM) and DeepSol employed convolutional neural network. When the models were evaluated on an independent test dataset of 2,001 *E. coli* proteins (1,000 soluble and 1,001 insoluble) (Chang et al., 2014), PaRSnIP achieved an accuracy of 0.74 and a MCC of 0.48, and DeepSol achieved an accuracy of 0.77 with an MCC of 0.55. One of PaRSnIP’s key advantages is its ability to provide feature importance scores, which allow researchers to identify sequence variants that are linked to solubility outcomes. For instance, proteins with higher fractions of exposed residues (FER) exhibited improved solubility, while tripeptides containing multiple histidines (IHH) were associated with a higher likelihood of insolubility (Rawi et al., 2018).

Moreover, PaRSnIP leverages GBM to model complex sequence-derived features efficiently, making it suitable for solubility prediction with moderate computational demands. However, its reliance on manually engineered features and the absence of structural data limit its generalizability, particularly for structurally complex proteins. DeepSol, utilizing a convolutional neural network, automates feature extraction, offering enhanced accuracy and adaptability across diverse datasets. Nonetheless, its dependence on sequence-based data without incorporating 3D structural information constrains its predictive capacity for proteins where solubility is driven by structural conformation.

**SoluProt :** SoluProt is also a classification model, trained by GBM using the dataset of 11,436 proteins (5,718 soluble and 5,718 insoluble proteins) built from TargetTrack database (Hon et al., 2021). The features used for the model development included compositions of amino acids and dipeptides, physicochemical properties, average flexibility, secondary structure content, average disorder, content of amino acids in transmembrane helices, and maximum identity to the *E. coli* PDB proteins.

In addition to these features, SoluProt employed an advanced feature selection process to prioritize properties highly correlated with protein solubility, particularly focusing on secondary structure and disorder content. The model also improved its robustness by filtering noisy training data, ensuring higher reliability in predictions and reducing the risk of erroneous outputs. This careful data curation and feature refinement allowed SoluProt to offer more precise predictions, even though its accuracy and MCC metrics were moderate. When SoluProt was evaluated on an independent test dataset of 3,100 proteins (1,550 soluble and 1,550 insoluble proteins) compiled from North East Structural Consortium (NESG) (Price et al., 2011), it achieved an accuracy of 0.59 and an MCC of 0.17. Despite these relatively modest metrics, SoluProt outperformed other classification models like PROSO II, DeepSol, and SOLpro in specific contexts, particularly due to its strong handling of noisy datasets.

SoluProt demonstrates a strong capacity for handling noisy datasets through its comprehensive feature selection process, which prioritizes attributes highly correlated with protein solubility, such as secondary structure and disorder content. For example, when SoluProt predictions were applied to a test set, selecting only the top 10% of sequences with the highest predicted solubility led to a 49.7% increase in the success rate of protein production. This ability to effectively filter and manage complex data enhances the model’s reliability in solubility predictions. However, despite these strengths, SoluProt has an overall predictive performance constrained by relatively modest accuracy and MCC values, which limit its applicability in high-precision solubility prediction tasks. Compared to more advanced models such as DeepSol and PROSO II, SoluProt may be less suited for applications requiring higher predictive accuracy.

**NetSolP :** NetSolP is a solubility classification model that leverages a transformer-based protein language model (Thumuluri et al., 2022). The model was trained on a curated dataset, primarily sourced from the PSI: Biology dataset, focusing on proteins expressed in *E. coli*. This training dataset comprised 12,216 proteins, with 66% reported as soluble (the exact proportion of insoluble proteins was unspecified). To ensure diverse representation, sequence identity partitioning was applied to reduce redundancy. NetSolP utilizes contextual embeddings generated from transformer-based models, which capture intricate relationships between amino acid residues. The model also integrates sequence profiles derived from multiple sequence alignments (MSAs), which assess amino acid conservation across homologous proteins. Additionally, physicochemical properties such as hydrophobicity and polarity further enhance the model's solubility prediction capabilities by combining both sequence-level and structural information. Additionally, a robust feature selection process is incorporated to reduce noisy data and mitigate biases in the dataset.

In terms of performance, NetSolP was evaluated on an independent test set of 1,323 proteins (620 soluble and 703 insoluble) and achieved an accuracy of 0.76 and an MCC of 0.40. This solid performance was demonstrated particularly for highly expressed *E. coli* proteins in the price dataset. Additionally, when applied to the Camsol mutation dataset, consisting of 19 proteins with 56 variants from various organisms (excluding *E. coli*) (Sormanni et al., 2015), NetSolP achieved an accuracy of 0.66.

NetSolP’s transformer-based architecture allows it to capture complex sequence-residue relationships critical for solubility prediction, offering broad generalizability across different protein sequences. However, the model’s moderate accuracy and MCC on challenging datasets highlight some limitations in precision. Moreover, the computational demands of the transformer-based architecture pose challenges in resource-limited settings, which affect its broader applicability.

**DeepSoluE :** DeepSoluE is a protein solubility classification model that uses a hybrid feature-based approach, combining physicochemical properties with distributed amino acid representation features through a Long Short-Term Memory (LSTM) network (Wang & Zou, 2023). The model was trained on a dataset of 11,436 *E. coli* proteins, evenly split between soluble (5,718) and insoluble (5,718) proteins. DeepSoluE incorporates a diverse set of features, including physicochemical properties such as isoelectric point, aromaticity, and molecular weight, alongside sequence embeddings derived from a Word2Vec representation of amino acids, utilizing a skip-gram model. Furthermore, the model integrates secondary structure content and sequence identity features, which enhance its predictive ability by capturing both detailed sequence information and broader physicochemical characteristics related to protein solubility.

During its independent evaluation on a dataset of 3,100 *E. coli* proteins (1,550 soluble and 1,550 insoluble), DeepSoluE achieved an accuracy of 0.59 and an MCC of 0.18, reflecting balanced performance in predicting both soluble and insoluble proteins.

While DeepSoluE effectively combines physicochemical properties and sequence representation features through its LSTM-based architecture, allowing for nuanced solubility prediction, its moderate accuracy and MCC suggest that it may not be the most suitable choice for high-precision tasks. As a result, its application might be limited in contexts where higher predictive accuracy is required.

**PROTSOLM :** PROTSOLM is a classification model developed for predicting protein solubility by integrating various data types, including protein sequence, structural information, and physicochemical properties (Tan et al., 2024). It utilizes a dataset known as PDBSOL-train, which consists of 58,138 proteins (30,419 soluble and 27,719 insoluble proteins) after removing redundancy at a 25% sequence identity cutoff. Notably, a significant portion of these proteins are expressed in *E. coli*.

The model employs a two-stage training approach: first, pre-training, followed by fine-tuning. In this process, protein sequences are encoded using the ESM2 framework, and local structural information is incorporated through equivariant graph neural networks (GNNs). This combination allows PROTSOLM to effectively capture the complex relationships between amino acids, enhancing its predictive capabilities.

For independent evaluation, PROTSOLM was evaluated on four external datasets: PDBSOL-test (3,230 proteins: 1,675 soluble, 1,555 insoluble), ESOL-agg (2,155 proteins: 951 soluble, 1,204 insoluble), NESG-SoluProt (1,784 proteins: 1,052 soluble, 732 insoluble), and NESG-DSResSol (3,640 proteins: 1,817 soluble, 1,823 insoluble). The ESOL-agg, NESG-SoluProt, and NESG-DSResSol datasets primarily feature proteins expressed in *E. coli*, while the PDBSOL-test dataset includes a mix of *E. coli*-expressed proteins and those from other expression systems. Specifically, it achieved an accuracy of 0.79 and an MCC of 0.58 on PDBSOL-test, 0.61 accuracy and 0.22 MCC on ESOL-agg, 0.61 accuracy and 0.23 MCC on NESG-SoluProt, and 0.60 accuracy with 0.21 MCC on NESG-DSResSol. This consistent performance highlights the model’s robustness and its ability to generalize across different datasets, making it a valuable tool for predicting protein solubility.

Overall, PROTSOLM advances the field of solubility prediction by combining various data modalities. Its use of GNNs allows for a more nuanced understanding of amino acid relationships, resulting in improved performance compared with models that rely solely on sequence data. However, the model’s reliance on high-quality structural data may pose limitation in scenarios where such information is not readily available, particularly in the early stages of protein design.

**PLM_Sol :** PLM_Sol is a classification model by integrating multiple protein language models (PLMs) to generate protein sequence embeddings. This model employs classification layers to improve solubility prediction (Zhang et al., 2024). It was trained on the Updated *E. coli* Solubility Dataset (UESolDS), which is curated from various sources, including TargetTrack, eSOL, and the PDB. This UESolDS includes 79,344 proteins, comprising 47,291 soluble and 32,053 insoluble proteins.

The PLM_Sol model utilizes attention-based algorithms to extract contextual embeddings from protein sequences, specifically leveraging ProtT5 and ESM2. It then applies a multilayer perceptron (MLP) for the classification of solubility. For evaluation, an external test set was created, consisting of 4,000 proteins (2,000 soluble and 2,000 insoluble proteins), selected randomly after filtering out sequences that exhibit more than 25% identity to the training data. When evaluated on this independent test set, PLM_Sol achieved an accuracy of 0.72 and an MCC of 0.46, representing a 9% improvement in F1 score compared to SoluProt and a 10.4% increase in MCC relative to EPSOL.

By leveraging modern protein language models, PLM_Sol significantly advances solubility prediction, enabling it to extract rich contextual embeddings from sequence data. This capability enhances the model’s ability to capture complex relationships between protein sequence and solubility, thus offering superior performance over earlier methodologies. However, the model's reliance on computationally intensive attention-based algorithms, coupled with its dependence on large datasets, may pose challenges in practical applications, particularly in environments with limited computational resources.

**SOLart :** SOLart is a regression model designed to predict quantitative solubility (Hou et al., 2020). The model was trained using the eSOL *E. coli* dataset, which was generated through high-throughput cell-free expression of *E. coli* ORFs, covering approximately 70% of the entire *E. coli* proteome (Niwa et al., 2009). From the eSOL proteins, those with experimental 3D structures in PDB or structural models in SWISS-MODEL were selected (Schwede et al., 2003; Waterhouse et al., 2018). Redundant sequences were removed using an identity cutoff of 25%, resulting in a selection of 406 proteins with quantitative solubility and 3D structures. The features used for the SOLart model included residue compositions, secondary structure content, protein size, solvent accessibility, statistical potentials, etc. The SOLart model was built using the random forest algorithm based on these features. For independent evaluation, solubility data from the eSOL *E. coli* dataset, excluded during training dataset preparation due to the identity cutoff, were prepared (550 proteins). Additionally, *S. cerevisiae* eSOL datasets were used as additional test sets, including 59 proteins with X-ray structures and 50 proteins with homology-modeled structures (Uemura et al., 2018). When evaluated on the three test datasets, SOLart demonstrated reliable performance across datasets from two different species, achieving R^2^ values of 0.448 on the *E. coli* test dataset, and 0.608 and 0.490 on the *S. cerevisiae* test datasets.

SOLart performs effectively in quantitative solubility prediction by integrating 3D structural data, making it highly suitable for proteins with well-characterized structures. Its random forest model efficiently combines structural and sequence features to deliver accurate cross-species predictions. However, its reliance on high-quality 3D structural data limits its applicability for proteins without experimentally determined or modeled structures, restricting its generalizability.

**Han et al.’s SVR model :** Han et al.’s SVR model is a regression-based model aimed at improving protein solubility by introducing optimized short peptide tags (Han et al., 2020). The model was trained on the *E. coli* eSOL dataset, which includes 3,148 proteins, using amino acid composition as input features. The SVR model predicts continuous solubility values. They refined peptide tags through a genetic algorithm to enhance their solubility properties. These peptide tags, typically 20 to 30 amino acids in length, are rich in aspartic acid (D) and glutamic acid (E), and improve solubility by increasing electrostatic repulsion between protein molecules, thereby preventing aggregation.

Experimental validation showed significant improvements in both solubility and enzymatic activity of modified enzymes. For example, the solubility of tyrosine ammonia lyase (TAL) more than doubled, and its enzymatic activity improved by 250%. Similar improvements were observed for other enzymes, such as 1-deoxy-D-xylulose-5-phosphate synthase (DXS), showing that the optimized tags not only enhance solubility but also improve protein folding quality, which in turn boosts the enzyme’s activity. This method presents a valuable tool for applications in metabolic engineering and other biotechnological fields (Hou et al., 2020).

**GraphSol :** GraphSol is another regression model designed to predict quantitative protein solubility (Chen et al., 2021). The *E. coli* eSOL dataset was split into 75% for training (2,052 proteins) and 25% for testing (685 proteins) (Niwa et al., 2009). GraphSol was trained by graph convolutional network algorithm utilizing sequence-derived features such as Hidden Markov model, PSSM, diverse physicochemical properties (e.g., steric parameters, hydrophobicity, volume, polarizability, isoelectric point), and structure-derived features such as relative solvent accessibility, backbone torsion angles, and protein contact information. When evaluated on the 685 *E. coli* proteins, it achieved an R^2^ of 0.483. As another evaluation, GraphSol was also evaluated on 108 *S. cerevisiae* proteins used for SOLart evaluation and the model achieved R^2^ of 0.37. GraphSol offers notable performance in integrating both sequence and structure-derived features, leveraging GCNs to capture complex relationships that influence protein solubility. Its ability to incorporate structural data provides an advantage in predicting quantitative solubility. However, its reliance on detailed structural information, like other structure-dependent models, limits its use in cases where such data is unavailable, potentially restricting its generalizability.

**CamSol :** CamSol is a physics-based computational model developed to improve protein solubility through rational design (Sormanni et al., 2015). Unlike machine learning models, which rely on training data to predict solubility outcomes, CamSol uses physicochemical principles such as hydrophobicity, charge distribution, and structural corrections to predict how specific mutations impact protein solubility. By rapidly screening thousands of potential mutations, CamSol identifies variants that improve solubility while maintaining the protein’s structural integrity and function.

CamSol has been successfully applied to design solubility-enhancing mutations for therapeutic proteins, particularly antibodies. One notable application was its use in predicting mutations for antibodies targeting the Alzheimer’s Aβ peptide, where experimental validation demonstrated a strong correlation between CamSol’s predictions and actual measured solubility in the lab. This makes CamSol a fast, cost-effective alternative for protein solubility prediction, with significant potential use in biotechnological and pharmaceutical industries.

**TISIGNER.com :** TISIGNER.com is an integrated platform that offers computational tools for optimizing recombinant protein production, addressing challenges such as low protein expression levels and solubility issues (Bhandari et al., 2021). The platform is particularly suitable for life science research and the development of biotherapeutics. Unlike traditional machine learning models, TISIGNER.com provided targeted solutions through three main tools: TIsigner, SoDoPE, and Razor.

First, TIsigner optimizes mRNA sequences to enhance protein expression by adjusting translation initiation site accessibility. This tool is customizable for various expression hosts, such as *E. coli* and *S. cerevisiae*, allowing researchers to achieve higher protein yields without labor-intensive trial-and-error approaches in the lab. Second, SoDoPE analyzes and optimizes protein solubility by identifying regions prone to aggregation, guiding mutagenesis experiments to improve stability during expression and purification. Lastly, Razor predicts signal peptides for protein secretion, ensuring proper translocation for secretory protein, and preventing intracellular accumulation and toxicity in host cells.

TISIGNER.com has been effectively used in applied research and industrial settings to improve recombinant protein production. TIsigner has optimized protein expression levels in large-scale production processes. SoDoPE has helped stabilize proteins during purification, and Razor has ensured correct translocation of secretory proteins, preventing harmful accumulation in host cells. By seamlessly integrating these tools, TISIGNER.com offers fast, efficient, and cost-effective solutions for protein production challenges, making it invaluable for biotechnology and pharmaceutical industries.

**Limitation of current models :** The computational models described in this review employ a variety of algorithms and datasets, providing a comprehensive exploration of different approaches to predict protein solubility. This diversity enables models to capture distinct patterns from the input data, thereby allowing for generalization across a wide spectrum of protein sequences. For example, models like PaRSnIP and DeepSol leverage different machine learning techniques such as gradient boosting machines and convolutional neural networks, respectively, which process features in unique ways. Despite the variation in algorithms and datasets, the overall predictive performance of these models, as measured by accuracy, MCC, and R2, does not exhibit significant fluctuations. Protein solubility is governed by a complex interplay of factors, many of which are not fully represented by the sequence- or structure-derived features typically used in current models. As a result, even with advancements in algorithms and dataset size, model performance tends to plateau. For classification models, accuracy scores typically range between 0.72 and 0.77, while MCC values tend to plateau around 0.5. For regression models, R² values typically fall between 0.4 and 0.6, reflecting the inherent difficulty in modeling the quantitative aspects of solubility.

To achieve substantial improvements in predictive performance, particularly beyond these thresholds, more diverse and comprehensive datasets are required. Additionally, the inclusion of higher-order features, such as structure-based properties, could further refine these models. However, until such datasets and features are widely available, significant advances in accuracy, MCC, or R² are unlikely, regardless of the algorithmic improvements.

## Challenges in the Development of Protein Solubility Prediction Models

The performance of machine learning models heavily relies on the quality and size of data used for training. Currently, over 80,000 qualitative solubility data were collected from various resources, but this dataset often contain inconsistent or unreliable data due to differences in experimental conditions, such as temperature and expression platform, and varying criteria for determining solubility.

This is a common issue in bioinformatics, where datasets are frequently sourced from diverse resources with different standards. To address the reliability and scalability issues associated with conventional experiments, high-throughput approaches based on cell-free protein synthesis systems have gained significant attention. The PURE system, for instance, enables the in vitro translation of mRNAs into proteins without the need for living cells, allowing for the production of recombinant proteins with minimal cellular contaminants (Doerr et al., 2021). This cell-free system facilitates high-throughput protein expression and solubility measurement.

However, despite its advantages, cell-free systems have limitations. In vitro and in vivo solubility can differ significantly due to the complex interplay between cellular components in vivo such as chaperones. For example, the study on the eSOL dataset found that only 32% of the expressed proteins were soluble (solubility >70%) (Delaney, 2004). Interestingly, when chaperones like DnaJKE or GroE were added to the in vitro expression system, 2/3 of the tested proteins showed a significant increase in solubility (de Marco et al., 2007; Niwa et al., 2012). This underscores the potential of chaperone-assisted co-expression to bridge the gap between in vitro and in vivo conditions. By mimicking the intricate cellular environments of in vivo systems, such approaches can greatly reduce discrepancies in protein solubility. Furthermore, optimizing in vitro conditions to replicate physiological environments—such as adjusting ionic strength, macromolecular crowding, and redox balance—can enhance the predictive accuracy of solubility models, ensuring that in vitro conditions align more closely with in vivo conditions. Given the importance of knowing solubility in vivo for recombinant protein production and function, the discrepancy between in vitro and in vivo solubilities is a significant challenge.

Another challenge is the skewed distribution of quantitative solubility data, which is similar to the imbalanced dataset problem encountered in classifier development (Thabtah et al., 2020). This issue is common in quantitative datasets (Kotronoulas et al., 2023). For instance, the eSOL dataset exhibits a bimodal solubility distribution, indicating that the data are biased towards highly insoluble and highly soluble proteins (Delaney, 2004). Such a skewed distribution can significantly affect the model's ability to accurately predict across the entire range of solubility. To address this, data augmentation techniques like the Synthetic Minority Over-sampling Technique (SMOTE) can be employed to balance the dataset by generating synthetic samples that represent underrepresented solubility classes (Kotronoulas et al., 2023). This approach enhances the distribution, enabling the model to learn more effectively across a broader range of solubility values and ultimately improving accuracy. Additionally, implementing stratified sampling during model training ensures a more even representation of solubility states, reducing overfitting and enhancing the model’s generalizability to unseen dataset. However, caution is needed with unconditional data augmentation methods, as they do not reflect actual experimental results.

Recently, high-performance models have utilized structure-based features from protein conformation (Jumper et al., 2021). While sequence-based features are appreciated for their simplicity and supported by extensive datasets (Hou et al., 2022), they frequently encounter challenges in accurately predicting solubility due to their inability to capture higher-order structures critical for solubility. Structure-based features, such as solvent accessibility, backbone torsion angles, and secondary structure content, offer more detailed information for solubility prediction, but they are limited by the availability of structure data and the computational demands required for their implementation. Despite recent breakthroughs in protein structure prediction methods (Jumper et al., 2021; Liu et al., 2022; Ruff & Pappu, 2021), which have enabled high-accuracy conformation predictions, these approaches remain computationally intensive and limit the practical application of solubility prediction models to identify optimal single amino acid mutations for protein engineering.

To overcome these challenges, hybrid models that combine machine learning techniques with physics-based solubility models could potentially enhance predictive accuracy by integrating both experimental and theoretical insights into protein behavior. Additionally, further refining the features related to protein structure and expression, such as integrating more detailed structural information from homology models or predicted protein structures using techniques like AlphaFold, could improve model reliability. As protein structure prediction tools continue to evolve, integrating these advancements into solubility prediction models will be essential for improving their accuracy and utility in bioindustrial applications.

## Conclusion

In summary, this review highlights the significant strides made in the field of protein solubility prediction through computational approaches. Despite these advancements, challenges still remain, particularly in the areas of data quality, the discrepancy between in vitro and in vivo solubility, and the skewed distribution of solubility data. The integration of advanced machine learning techniques with both sequence-based and structure-based features promises to improve predictive accuracy. As protein structure prediction technologies continue to evolve, these high-accuracy models will facilitate the design of proteins with desired solubility characteristics. These developments will contribute to more efficient and scalable protein engineering processes, benefiting various applications in biotechnology and related industries.

## Figures and Tables

**Table 1.** Overview of protein solubility prediction models: dataset information, algorithm type, features, performance, and pros and cons

Model		Dataset (soluble + insoluble)	Machine learning algorithm	Features	Performances	Pros	Cons	References
Classification models	PROSO	*E. coli*	Chained models of SVM and naïve Bayes	Amino acid compositions and alpha-helical structure composition	Crossvalidation:	Computationally efficient, simple to use	Lacks structural data, limited in complex solubility cases	Smialowski et al. (2007)
		> 14,000			Accuracy: 0.72			
		(50% and 50%)^1)^			AUC: 0.78			
		Model is not available to access						
	SOLpro	*E. coli*	Chained 20 SVM models with 1 SVM output model	Compositions (amino acids, dipeptides, and tripeptides), physicochemical properties (hydropathy, charge, molecular weight, aliphatic index, etc.), secondary structure composition, exposed residues, number of domains, etc.	Crossvalidation:	Advanced SVM-based architecture, better accuracy	Constrained by sequence-only features, no structural insight	Magnan et al. (2009)
		17408			Accuracy: 0.74			
		(8,704+8,704)			AUC: 0.74			
		Web-based tool is available at https://scratch.proteomics.ics.uci.edu/						
	PROSO II	*E. coli*	Chained model: two input models of Parzen window model and logistic regression classifier, and an output model of logistic regression classifier	Compositions (amino acids, dipeptides, and tripeptides), physicochemical properties (isoelectric point, GRAVY index, etc.), secondary structures, exposed residues, and number of domains	Independent test:	Improved with larger datasets and refined algorithms	No structural data, reduced accuracy for complex proteins	Smialowski et al. (2012)
		82,299^2)^			Accuracy: 0.75			
		for training						
		1765						
		(⅙ + ⅚)^1)^						
		hold-out for test						
		Model is not available to access						
	PaRSnIP	*E. coli*	Gradient boosting machine	Compositions (amino acids, dipeptides, and tripeptidmagnes), sequence length, molecular weight, fraction of turn-forming residues, average hydropathicity, aliphatic index, absolute charge, secondary structures, hydrophobicity of exposed residues.	Independent test:	Effective use of GBM for feature handling	Relies on manual feature selection, no structural data	Rawi et al. (2018)
		69420			Accuracy: 0.74			
		(28,972+40,448) for training			MCC: 0.48			
		2001						
		(1,000+1,001)^3)^ for testing						
		Source codes are available at https://github.com/RedaRawi/PaRSnIP						
	DeepSol	*E. coli*	Convolutional neural network	Amino acid compositions, molecular weight, absolute charge, aliphatic index, average hydropathicity (GRAVY), fraction of turn-forming residues, secondary structures, fraction of exposed residues, and hydrophobicity of exposed residues.	Independent test:	Automated feature learning with higher accuracy	Lacks structural data, limited for complex proteins	Khurana et al. (2018)
		69420			Accuracy: 0.77			
		(28,972+40,448) for training			MCC^4)^: 0.55			
		2001						
		(1,000+1,001)^3)^ for testing						
		Source codes are available at https://zenodo.org/records/1162886						
	SoluProt	*E. coli*	Gradient boosting machine	Compositions (amino acids and dipeptides), physicochemical properties, average flexibility, secondary structure content, average disorder, residue content in transmembrane helices, maximum identity to *E. coli* proteins in PDB	Independent test:	Robust handling of noisy data, effective feature selection	Lower accuracy and MCC, less suited for high-precision tasks	Hon et al. (2021)
		11436			Accuracy: 0.59			
		(5,718+5,718) for training			MCC: 0.17			
		3100						
		(1,550+1,550) for testing						
		Web-based tool and datasets are available at https://loschmidt.chemi.muni.cz/soluprot/						
	NetSolP	*E. coli*	Two input models, ESM1b and ProtT5 transformer-based models, with an output passed to a classification layer	Sequence embeddings (from transformer models like ESM1b and ProtT5), sequence profiles (MSAs generated using HHblits), and amino acid conservation (calculated using conservation scores).	Independent test:	Transformer-based model captures complex sequence-residue interactions	Moderate accuracy and MCC, computationally demanding	Thumuluri et al. (2022)
		12216			Accuracy: 0.76			
		(66% + 34%)^1)^ for training			MCC: 0.40			
	
		1323						
		(620+703) for testing						
		Source codes are available at https://github.com/TviNet/NetSolP-1.0						
	PROTSOLM	*E. coli*	Multi-modal model: two input models, ESM2 for protein sequence embedding and equivariant graph neural networks (EGNNs) for structural feature encoding, combined with an output model of a deep learning classifier. Gradient boosting machine	Sequence embeddings (ESM2-650M), inter-residue distances, backbone geometry, physicochemical properties (charged residues, GRAVY index, and turn-forming residues), secondary structure content, solvent accessibility, hydrogen bond density, hydrophobicity of exposed residues, and structure confidence (pLDDT from ESMFold).	Independent test:	Multimodal approach integrates sequence and structure data	Dependence on structural data may limit broad applicability	Tan et al. (2024)
		64598			Accuracy: 0.79			
		(33,763+30,835) for training			MCC: 0.58			
		3230			Independent test:			
		(1,675+1,555) for testing			Accuracy: 0.60			
					MCC: 0.22			
		2155			Independent test:			
		(951+1,204)^6)^ for testing			Accuracy: 0.60			
					MCC: 0.23			
		1784						
		(1,052+732)^6)^ for testing						
		3640			Independent test:			
		(1,817+1,823)^6)^ for testing			Accuracy: 0.60			
					MCC: 0.21			
		Source codes are available at https://github.com/tyang816/ProtSolM						
	PLM_Sol	*E. coli*	Two input embedding models, ProtT5 and ESM2, are combined with an output model of biLSTM_TextCNN layer	Protein sequence embeddings (capturing contextual information such as residue-level interactions and sequence structure).	Independent test:	Leverages PLMs for richer contextual embeddings	Computationally intensive, dependent on large datasets	Zhang et al. (2024)
		79344			Accuracy: 0.72			
		(47,291+32,053) for training			MCC: 0.46			
		4000						
		(2,000+2,000) for testing						
		Source codes are available at https://zenodo.org/records/12881509						
	DeepSoluE	*E. coli*	Long Short-Term Memory network	Physicochemical properties (isoelectric point, aromaticity, molecular weight, flexibility, and instability index), sequence embedding, and secondary structure content, along with structural-based features (protein sequence length, residue-level solvent accessibility, and torsion angle domain).	Independent test:	Balanced approach, integrates physicochemical features	Moderate accuracy and MCC, less suited for high-precision tasks	Wang & Zou (2023)
		11436			Accuracy: 0.59			
		(5,718+ 5,718) for training			MCC: 0.18			
		3100						
		(1,550+1,550) for testing						
		Web-based tool and datasets are available at http://lab.malab.cn/~wangchao/softs/DeepSoluE/						
Regression models	SOLart	*E. coli *	Random forest	Compositions of amino acids, secondary structure content, protein length, protein solvent accessibility, and statistical potentials (residue-level solvent accessibility and torsion angle domain).	Independent tests:	Accurate for quantitative solubility predictions, strong cross-species performance	Limited by dependence on 3D structural data	Hou et al. (2020)
		406^5)^ for training			on *E. coli*			
					R^2^: 0.448			
		*E. coli*			RMSE: 23%			
		550^5)^ for testing			on *S. cerevisiae*			
					R^2^: 0.608, 0.490			
		*S. cerevisiae*			RMSE: 23%, 20%			
		59 and 50^5)^ for testing						
		Web-based tool is available at http://babylone.ulb.ac.be/SOLART						
	SVR Model	*E. coli*	Support Vector Regression	Compositions of amino acids	Independent tests:	Efficient solubility optimization, successful experimental validation and versatile applicability	No internal test dataset and limited consideration of stability	Han et al. (2020)
		3,148^5)^ for training			on *E. coli*			
					R^2^: 0.57			
		4 proteins^5)^						
		for experimental validation						
		Source codes are available at https://github.com/KangZhouGroupNUS/optimization_protein-solubility						
	GraphSol	*E. coli*	Graph convolutional network	Hidden Markov model, PSSM, diverse physicochemical properties (steric parameters, hydrophobicity, volume, polarizability, isoelectric point, etc.), relative solvent accessible surface area, backbone torsion angles, protein contact map, etc.	Independent tests:	Strong integration of sequence and structure	Dependent on structural data, limiting generalizability	Chen et al. (2021)
		2052^5)^ for training			on *E. coli*			
					R^2^: 0.48			
		*E. coli*			on *S. cerevisiae*			
		685^5)^ for testing			R^2^: 0.37			
	
		*S. cerevisiae*						
		108^5)^ for testing						
		Source codes are available at https://github.com/jcchan23/GraphSol						

^1)^ Only percentages or ratios have been reported.

^2)^ The ratio of soluble and insoluble data has not been reported.

^3)^ External qualitative solubility dataset from the study of Chang et al. (2014).

^4)^ Mathew’s correlation coefficient (MCC), a balanced accuracy for imbalanced dataset.

^5)^ Quantitative solubility datasets for regression model training and testing.

^6)^ External qualitative solubility dataset from literature of Tan et al., Niwa et al., and Smialowski et al., respectively (Niwa et al., 2009; Smialowski et al., 2012; Tan et al., 2024).

**Table 2.** Feature generation tools

Tool name	Number of features	Feature	URL	References
PROFEAT	< 2,000	Residue compositions, physicochemical properties, sequence order and secondary structures, topological characteristics, interaction patterns, and other network properties	http://bidd2.nus.edu.sg/cgi-bin/profeat2016/main.cgi ^*^	Zhang et al. (2017)
iFeatureOmega	> 18,000	Residue compositions, physicochemical properties, sequence order and secondary structures, half sphere exposure, residue depth, atom composition and network-based index	https://github.com/Superzchen/iFeatureOmega-CLI	Chen et al. (2022)
protr	22700	Residue compositions, physicochemical properties, secondary structure, similarity score, customizable descriptors (AAindex database), Auxiliary functions	https://github.com/nanxstats/protr	Xiao et al. (2015)
Rcpi	> 10,000	Residue composition, physicochemical properties, secondary structures, PSSM profile, PCM, GO similarity, sequence similarity. Rcpi also provides compound-related features and protein-compound/protein-protein interactions features	https://github.com/nanxstats/Rcpi	Cao et al. (2015)
Propy	9547	Residue compositions, physicochemical properties, sequence order coupling numbers, pseudo amino acids compositions.	https://github.com/MartinThoma/propy3	Cao et al. (2013)
PDBparam	> 50	Physicochemical properties, secondary structures, inter-residue interactions, identification of binding sites from PDB structure	https://www.iitm.ac.in/bioinfo/pdbparam/index.html	Nagarajan et al. (2016)
POSSUM	12010	PSSM-based features	https://possum.erc.monash.edu/	Wang et al. (2017)
Pfeature	200,000+	Diverse sequence-based features, binary profiles, evolutionary information based on PSSM, structural features, and pattern-based features	https://github.com/raghavagps/Pfeature	Pande et al. (2023)

*Not accessible at the time of manuscript preparation.
